# Socio-economic determinants of household out-of-pocket payments on healthcare in Pakistan

**DOI:** 10.1186/1475-9276-11-51

**Published:** 2012-09-04

**Authors:** Ashar Muhammad Malik, Shah Iqbal Azam Syed

**Affiliations:** 1 Community Health Sciences Department, Aga Khan University, Stadium Road, Karachi, 78400, Pakistan; 2 Community Health Sciences Department, Aga Khan University, Karachi, Pakistan

**Keywords:** Out-Of-Pocket payment, Social and economic determinants, Equity, Healthcare financing, Demand for health, Health policy, Developing countries, Pakistan

## Abstract

**Background:**

Out-of-pocket (OOP) payment on healthcare is dominant mode of financing in developing countries. In Pakistan it is 67% of total expenditure on healthcare. Analysis of determinants of OOP health expenditure is a key aspect of equity in healthcare financing. It helps to formulate an effective health policy. Evidence on OOP in Pakistan is sparse. This paper attempts to fill this research gap.

**Methods:**

We estimated determinants of OOP payments on healthcare in Pakistan. We used data sets of Pakistan Household Integrated Economic Survey (HIES) and Pakistan Standard of Living Measurement (PSLM) Survey for the year 2004-05. We developed a multiple regression model for the determinants of OOP payments using methods of Ordinary Least Square (OLS). We mainly used social, economic, demographic and health variables in our analysis.

**Results:**

Median household OOP healthcare in the year 2004-05 was Pakistani Rupees (PKR) 2500 (US$ 41.99) in 2004-05. Household non-food expenditure was the single highest significant predictor of household OOP health expenditure. Household features like literate head and spouse, at least one obstetric delivery in last three years, unsafe water, unhygienic toilet and household belonging to Khyber Pukhtonkhwa (KPK) province were significant positive predictors of OOP payments. Households with male head, bricks used in housing construction, household with at least one child and no elderly, and head of household in a white collar profession were negative predictors of OOP payments.

**Conclusion:**

Our analysis confirms earlier findings that economic status and number of old aged members are significant positive predictors of OOP payments. This association can direct government to enhance allocations to healthcare and to include program focusing on non-communicable diseases. Our findings suggest further research to explore beneficiaries of government healthcare programs and determinants of high OOP payments by household residing in KPK province than other province. The interaction between white collar profession and their economic status in predicting OOP payments is also an area for further research.

## Background

Out-of-pocket (OOP) payment is the dominant mode of financing healthcare in developing countries [1]. In the case of OOP payments, accessing healthcare services is dependent on the economic status of the individual or household. Meeting demand for healthcare is a great challenge if the cost is unaffordable [2]. Households may borrow money, sell assets or divert resources from other needs to seek healthcare. They may opt for less costly traditional or sub-optimal care, or altogether forgo healthcare services they need [3]. Thus an out-of-pocket payment is considered the most inequitable financing mechanism [4].

One of the main objectives of national and international health policy is to replace OOP payments with more equitable modes of financing [5]. In this context analysis of determinants of OOP payments is important for devising an effective health policy. The role of socio-economic, geographic and environmental, lifestyle and other factors is well documented in determining health and health seeking behavior [6]. This argument draws from the seminal work of Michael Grossman on health production and the demand for health, i.e. multiple factors contribute to health [7]. The behavioral model of health service-use also emphasizes role of multiple factors in determining health services-use such as demography, social structure and health beliefs, availability of health services, financial resources and community support, perceived and actual need for healthcare and consumer satisfaction [8,9].

Like many developing countries OOP payment is dominant mode of financing healthcare in Pakistan. The per capita total expenditure on health is US$26. The share of OOP payment was 67% of total expenditure on health and 85% of private expenditure on health in the year 2010 [10]. People pay out-of pocket for treatment in private hospitals and clinics as-well-as unofficial payments for healthcare at the government facilities.

Pakistan is a federation of four provinces. It currently has a population of 177 million. This population is mainly rural with a recent trend towards urbanization. Currently the per capita income is US$1,254 [11]. Government has recognized financial protection for healthcare as major objective of its social protection policy [12]. Evidence on household demand for healthcare, social determinants of health and the nature and extent of out of-pocket payments is direly needed to supplement government efforts to devise and effective health policy in Pakistan. We found few scientific research papers on healthcare financing in Pakistan. These papers mainly cover government health expenditure [13,14]. The purpose of this paper is to help fill this gap in health services research and contribute to improved health policy in Pakistan by estimating the determinants of household OOP payments in Pakistan.

## Methods

### Data

We used the data set of the Pakistan Living Standard Measurement (PSLM) Survey for 2004-05. It is a nationally representative survey covering all four provinces of the country. The survey collected data on various aspects of social and living standards. It included demography, health, education, water supply & sanitation, housing, household income and expenditures, and public services use and satisfaction. The core welfare indicator approach is used in this survey [15].

Sample size of PSLM was 76,520 households. The Household Integrated Economic Survey (HIES) was carried out on a sub-sample of 14,708 households. The respondents in the PSLM survey were all individuals in the households, but in the HIES it was solely head of the households. The sampling technique of both the surveys was multi-stage cluster sampling with stratification. Sampling weights were also provided in the survey. The HIES, being a sub-sample of PSLM, provides a unique opportunity to associate income and expenditure patterns of households with their social and demographic and other characteristics from the PSLM.

Out-of-pocket payment on healthcare was reported in the “consumption of consumable goods and services” module of the survey. It was reported in four different categories i.e.

 1) Purchase of medicines, equipment supplies etc.

 2) Medical fees paid to doctors, Hakeem(traditional healer) etc. outside hospital including medicines

 3) Hospitalization including doctors’ fees, laboratory tests, X-ray charges etc. and

 4) Dental/optical care and all other expenses on healthcare not classified elsewhere.

The recall period of health expenditure was one year.

### Variables

We mainly used household level characteristics. Certain individual characteristics that can influence OOP payments, e.g. some characteristics of head and spouse in a household are also included in our analysis. All independent variables included in our analysis mainly covered economic, demographic, social and living standard, and health characteristics of the households

#### Economic

We used household non-food expenditure as a proxy of economic status of a household. Due to the positively skewed distribution of non-food expenditure we used its natural logarithm transformation.

We transformed the profession of the head of household to the lifestyle dummy variables. We assumed that a household is living a modern lifestyle if its head is in the professional categories of senior officials/managers and professionals.

#### Demographic

We included the educational level of the head and spouse of the household as a predicator for OOP payments. Five years of formal schooling is considered as literate. For provincial differences in OOP payments four dummy variables for provinces of Punjab, Sindh, Khyber Pukhtonkhawa (KPK) and Baluchistan were included in the analysis.

The household members aged more than 60 years and less than five years were assumed to be major predictors of OOP payment compared to other members. The four dummies were created for households with

 1) No children or elderly

 2) At least one child and no elderly

 3) At least one elderly and no children and

 4) At least one child and one elderly in the household.

The gender of the head of household was also included in our analysis.

#### Social and living standard

A dummy variable of bricks as the housing construction material is included to explore influence of housing on OOP payments compared to other less durable housing materials.

Drinking water source and types of toilets were also transformed into a dummy predictor of OOP payments. We assumed all types of water source of the household as unsafe except “piped water inside the house”. All types of toilet facilities were assumed unsafe except “flush to public sewerage”.

#### Health and healthcare

Households reported the time and distance, and mode of travelling, to reach a nearby health facility. We assumed that distance to reach hospital and clinics could potentially predict the OOP payment in the form of traveling cost. The number of obstetrical deliveries in the last three years in a household was transformed to categorical variable of any delivery in last three years in the household.

### Statistical analysis

We used Ordinary Least Square (OLS) methods to estimate a multiple linear regression model of OOP payments. Household OOP payments are usually characterized as positive, with sizable zero responses and a positively skewed distribution of the data [16]. In case of the HIES data, 14,488 households (98.5%) reported OOP payments. It exhibited a positively skewed distribution. We applied a natural logarithm transformation of health expenditure in the regression model.

We explored the multi-colinearity of the independent variables. We estimated the variance inflation factor (VIF). Its value remained less than 3.41 predicting limited multi-colinearity.

We reported our estimates in median, inter-quartile range, and percentage. All analysis was carried out in STATA version 11.2, Stata Corporation, Texas, USA, 2011.

## Results

### Descriptive analysis

Median household OOP payment on healthcare in the year 2004-05 was Pakistan Rupees (PKR) 2500 (US$ 41.99). It was highest in Khyber Pukhtonkhwa (KPK) (PKR 3000, US$ 50.39). Median OOP payment on medicine was highest in KPK (PKR 2400, US$ 40.32). Median OOP payment on hospitalization was highest in Punjab (PKR 1000, US$ 16.80).

We found marked differences in household OOP payment related to their socio-economic characteristics. Households with both head and spouse literate, urban households and households with at least one obstetric delivery in last three years reported higher OOP payments than the other households.

In half of the households the heads and spouses were illiterate (50.9%). The literacy of household head and spouse was higher in Punjab and Sindh provinces than in other two provinces. The majority of the households were headed by a male in all four provinces with KPK being the lowest in this ranking. The detail of the descriptive analysis is given in Table 1.

**Table 1:** Descriptive statistics of determinants of out of pocket payments in Pakistan

**Household characteristics**	**Provinces**				**National**
	**Punjab**	**Sindh**	**KPK**	**Baluchistan**	
Household OOP payment (PKR)	1800 (3000)	2500 (2500)	3000 (4150)	2500 (2300)	2500 (2990)
Household non-food expenditure (PKR)	22050 (39080)	19045 (21130)	20945 (25350)	22100 (19140)	21120 (24674)
Modern life style	7%	12%	7%	8%	8%
Literacy of head and spouse					
-Both head and spouse are illiterate	2895 (39%)	1560 (21%)	1732 (23%)	1291 (17%)	7478 (51%)
-Only spouse is literate	178 (70%)	47 (19%)	23 (9%)	5 (2%)	253 (2%)
-Only head is literate	1401 (36%)	1042 (27%)	760 (20%)	673 (17%)	3876 (26%)
-Both head and spouse are literate	1632 (53%)	828 (27%)	446 (14%)	178 (6%)	3084 (21%)
Population (millions)	63.92 (41.56%)	36.43 (23.67%)	31.03 (20.16%)	22.48 (14.61%)	153.92 (100%)
Rural Population (millions)	37.71 (59%)	20.76 (57%)	19.55 (63%)	15.06 (67%)	92.35 (60%)
Male head of the household	5584 (91%)	3396 (98%)	2570 (87%)	2117 (98%)	13667 (93%)
Household Size	4 (3)	4 (2)	6 (4)	7 (3)	5 (3)
Children (< 5 years) & elderly (> 60 years)					
-No children or elderly	2077 (43%)	1154 (24%)	835 (17%)	718 (15%)	4784 (28%)
-At least one elderly and no children	1042 (51%)	430 (21%)	333 (16%)	236 (12%)	2041 (14%)
-At least one child and no elderly	2026 (35%)	1506 (26%)	1244 (21%)	998 (17%)	5774 (39%)
-At least one elderly and one child	961 (46%)	387 (18%)	549 (26%)	195 (9%)	2092 (14%)
Unhygienic toilet facility	4694 (39%)	2581 (21%)	2769 (23%)	2038 (43%)	12082 (82%)
Unsafe drinking water	4192 (47%)	2029 (23%)	1597 (18%)	1144 (56%)	8962 (61%)
Housing material (bricks)	3594 (41%)	2178 (25%)	1656 (19%)	1377 (16%)	8805 (60%)
Any obstetric delivery in last 3 years in the household	2481 (38%)	1615 (25%)	1520 (23%)	935 (14%)	6551 (45%)
Health facilities distance constraint	1774 (33%)	1574 (29%)	969 (18%)	1060 (20%)	5377 (37%)

*Percentage sign (%) is mentioned where numbers and percentages are given, otherwise median and inter quartile range.

### Econometric analysis

We considered all variables in the multiple linear regression models that were significant predictors of OOP payments in the uni-variate analysis.

Non-food expenditure (LOG_NFE) emerged as the largest significant predictor of the log of OOP payment. An increase of 0.769 in the log of OOP payment in Pak Rupees was associated with a unit increase in the log of non-food expenditure in Pak Rupees. This was followed by households in KPK province, literate households and houses with unhygienic toilets. Details of the regression model are given in Table 2.

**Table 2:** Estimated coefficients in the OLS linear regression model for OOP payment in Pakistan

**Independent Variables**	**Coefficient**	**95% confidence intervals**	
Log of Non-food-expenditure	0.769	0.737	0.800
Modern life style	−0.092	−0.164	−0.020
Literacy of head and spouse			
-Both head and spouse are illiterate	-	-	-
-Only spouse is literate	0.166	0.104	0.228
-Only head is literate	0.136	0.005	0.266
-Both head and spouse are literate	0.233	0.172	0.293
Male head of household	−0.107	−0.184	−0.030
Household with children aged less than five years and elderly aged 60 years or above			
-No children or elderly	-	-	-
-At least one elderly and no children	0.066	−0.005	0.137
-At least one child and no elderly	−0.016	−0.084	−0.051
-At least one elderly and one child	0.107	0.029	0.185
Provinces			
-Punjab	-	-	-
-Sindh	0.102	0.059	0.0145
-KPK	0.402	0.351	0.453
-Baluchistan	0.037	−0.011	0.084
Unhygienic toilet facility	0.182	0.124	0.240
Unsafe drinking water	0.096	0.052	0.139
Housing material (bricks)	−0.026	−0.064	0.013
Any obstetric delivery in last 3 years in the household	0.179	0.116	0.242
Health facilities distance constraint	0.064	0.023	0.104
Constant	−0.419	−0.774	−0.065

Dependent variable log of out of pocket payments.

Total observation 14708, censored observation =12725, F (17,12707) = 186.37.

R^2^ = 0.3768, Root MSE = 0.78336.

Ramsey Reset Test F (3, 12704) =4.10, p = 0.0064.

Literate head and spouse of the household, urban households, unsafe water source, houses with unhygienic toilets and households with at least one child and one elderly person were significant predictors of OOP payments. Households where the head is in a white collar profession and male heads of the household negatively predicted OOP payments. Households being at a greater distances from health facilities was a positive predictor of higher OOP payments than OOP payments by households taking less than 30 min to reach a hospital or clinic.

## Discussion

The influence of socio-economic and other factors on health is well recognized. Our analyses of the situation in Pakistan are the first of its kind to be published in Pakistan. We used OLS methods for analysis of OOP payments which is appropriate if there are fewer zero values of OOP payments by the household. We considered the Matsaganis and Mitrakos et al. (2009) argument on choice of econometric model for OOP payments appropriate for our analysis. Their sampling unit and recall period of health expenditure was similar to our data set, i.e. household and one year recall period. A one year recall and aggregate health expenditure for the entire household would likely reveal fewer zero responses. The authors compared log OLS linear regression, two parts regression and generalized linear regression to model OOP payments. They reported similar results to alternative estimators [17].

We provided some useful additional information which increases the understanding of the determinants of OOP payments in Pakistan. We adapted various determinants of OOP payments from the literature review to our model considering data availability and our understanding of socio-economic and cultural factors in Pakistan. For some determinants of OOP payments, our findings confirmed earlier research, such as income, age and schooling. While for other determinants we opened a new debate on research into the determinants of OOP payments. Positive predictors of OOP payments in our model, e.g. household income or expenditure support earlier research finding [18-20]. The economic status in these research papers was the log of income, log of expenditure and household animal value, and wealth index respectively. In our model the log of non-food expenditure significantly predicted household’s OOP payments. It confirms the argument that non-food expenditure is an appropriate proxy of household effective income that predicts OOP payments better than total income or total expenditure [21].

In our model the greater influence of households with elderly members on OOP payments compared to households with children was similar to the findings of Tin-Su and Pakhrel et al. (2006) i.e. adult respondent as positive predictors (regression coefficient 0.439) of OOP payments than young [19]. Rous and Hotchkiss (2003) reported positive age related influences on OOP payments of all age groups except age 1-14 years. This effect was highest for respondents in age group of 65 years and above (regression coefficient 0.651) [18]. The lack of health sector resource allocation by the government for management of non-communicable diseases in the elderly could be a possible explanation of the positive influence of a household member of 60 and older on OOP payments in Pakistan.

Our findings on literacy of the head of household as positive predictor of OOP payments was similar to Tin-Su and Pakhrel et al. (2006) and Okunade and Suraraetdecha et al. (2009) [19,20]. Rous and Hotchkiss (2003) found it to be a negative predictor of OOP payments [18]. Our findings show that urban households made higher OOP expenditures on healthcare than rural households. This contradicted Rous and Hotchkiss’s (2003) findings [18].

White collar households are a negative predictor of OOP payments in our analysis. It is contradictory to the income and health expenditure relationship discussed above. We could not find any research article that has included profession of the head of household in the analysis of determinant of OOP payments. Fair access to free healthcare at government facilities and healthy life style could be possible explanations. Earlier research found that government subsidies in health sector in some developing countries for instance Nepal, China, Indonesia and India, benefited the rich more than the poor [22,23]. However this conclusion is based on income/expenditure of the household rather the profession or lifestyle.

One important aspect from the health policy perspective is the provincial differences in OOP payments. Our regression analysis indicated that a household in KPK province was higher predictor of OOP payment than for households in Punjab. Khyber Pukhtonkhwa province is generally considered to be a more conservative society with a predominance of population of Pushtoon ethnicity. It has a greater rural population, lower literacy, lower level of sewerage systems and larger household size than the other provinces. In KPK province more female heads of household than other provinces. In the regression model male head predicted negative influence on log of OOP than female heads. This finding is contrary to Rous and Hotchkiss (2003) findings regarding influence of male head of households on OOP payments. Besides other determinants we can associate higher OOP payments in KPK to the more households headed by a female than other province.

We only extracted that data which was attributed to households rather than individuals. In many cases there was a heterogeneity issue of individual characteristics and behavior within a household. For example individual satisfaction with services, vaccinations, etc. could not be grouped into a single indicator for households. Unlike Rous and Hotchkiss (2003) [18], we did not include a providers’ choice variable in our model since the unit of analysis in their data was the individual. In case of the HIES data, the unit of analysis was households. The heterogeneity of such provider choices within the household could not allow provider’s choice to be used as a predictor in the regression model. We grouped provider choices into public and private providers in the regression model but it was insignificant in explaining the variation in OOP payments (i.e. regression coefficient was-0.01) Unlike Tin Su and Pokhrel et al. (2006) [19] we could not use morbidity and mortality in our analysis due to limited data in PSLM survey.

The double hurdle model of Okunade and Suraraetdecha et al. (2009) was a time series analysis of determinants of health expenditure. They used permanent income instead of current income or expenditure to compare trends over many years 1994, 1996, 1998 and 2000 [20]. In our case, the analysis was carried out on OOP payments reported for one year. This kind of time series research would be worth undertaking in Pakistan in future, but only when similar data is made available for later years.

The comparison of our results with earlier research is not conclusive. These comparisons are constrained by differences in the selection of econometric model, the choice of independent variables and their interaction. There are also significant differences in the time period and sampling unit among the research studies reviewed, i.e.one time [17-19] or time series[20] and sampling unit of data collection i.e. individual [18,19] or households [17,20].

Findings of our research can help devising more effective health policy in Pakistan. Government should consider enhancing resources to healthcare. It is equally important for the government to understand beneficiaries of public provision of healthcare services. Parallel to government spending other prudent and sustainable risk pooling mechanism can help reducing intensity of OOP payments. Enacting mandatory social health insurance legislation can be a suitable option in the backdrop of rapid economic growth in the country since last decade. Thirdly non-communicable diseases require particular attention of the government in resource allocation priority settings in to health sector in Pakistan. Our findings also encourage provincial level analysis of OOP-Payments, especially in KPK such analysis are important from health policy perspective.

## Conclusion

Our findings strengthen the argument that multiple factors influence OOP payments. It supplemented the approach of Anderson that the “family as a unit” affects the choices regarding the seeking healthcare. We added some new elements to determinants of OOP payments in Pakistan. We included literacy of household head and spouse in our model and assessed their joint influence on OOP payments. The influence of the profession of the head of household on OOP payments is another important innovation in our analysis that should be further explored theoretically and empirically. We have provided important new information relevant to policy on OOP payments in Pakistan that will support improved health policy and programs in future.

## Competing interests

None.

## Authors’ contributions

MAM did the literature search and review, develop the econometric model and writing the first draft. SIA did the data cleaning, model specification, reviewing the results and the manuscript. All authors read and approved the final manuscript.

## References

[B1] O’Donnell O van Doorslaer E Rannan-Eliya RP Somanathan A Adhikari SR Akkazieva B Harbianto D Garg CC Hanvoravongchai P Herrin AN Huq MN Ibragimova S Karan A Kwon S Leung GM Lu JR Ohkusa Y Pande BR Racelis R Tin K Tisayaticom K Trisnantoro L Wan Q Yang B Zhao Y Who pays for health care in Asia? J Health Econ 2008 27 460 475 10.1016/j.jhealeco.2007.08.005 18179832

[B2] The World Health Organization The World Health Report – Health systems financing: The path to universal coverage 2010 2010 The World Health Organization, Geneva http://www.who.int/whr/2010/en/index.html 10.2471/BLT.10.078741 PMC2878164 20539847

[B3] Goudge J Russell S Gilson L Gumede T Tollman S Mills A Illness-related impoverishment in rural South Africa: why does social protection work for some households but not others? J Int Dev 2009 21 231 251 10.1002/jid.1550

[B4] Wagstaff A van Doorsaler E Culyer AJ, Newhouse JP Equity in healthcare financing and delivery Chapter 34 in Handbook of health economics Volume B 2000 1 Elsevier, North Holland 1803 1862

[B5] The World Health Organization World Health Report: Improving health systems performance 2000 2000 The World Health Organization, Geneva http://www.who.int/whr/2000/en/

[B6] Marmot M Friel S Bell R Houweling TA Taylor S Commission on Social Determinants of Health Closing the gap in a generation: health equity through action on the social determinants of health Lancet 2008 372 1661 1669 10.1016/S0140-6736(08)61690-6 18994664

[B7] Grossman M On the concept of health capital and the demand for health J Polit Econ 1972 80 223 255 10.1086/259880

[B8] Anderson MR Bartkus DE Choice of medical care: a behavioral model of health and illness behavior J Health and Soc Behav 1973 14 4 348 362 10.2307/2136779 4773924

[B9] Anderson MR Revisiting the behavioral model and access to medical care: does it matter? J Health and Soc Behav 1995 36 1 10 10.2307/2137284 7738325

[B10] The world health organization global health observatory data repository: country statistics Pakistan http://apps.who.int/ghodata/?vid = 15300&theme = country

[B11] Ministry of Finance Pakistan Economic Survey 2011 Government of Pakistan, Islamabad http://www.finance.gov.pk/survey_1011.html

[B12] Planning Commission Pakistan Framework for Economic Growth 2011 Government of Pakistan, Islamabad www.pc.gov.pk/hot%20links/growth_document_english_version.pdf

[B13] Siddiqui R Afridi U Haq R Determinants of expenditure on health in Pakistan Pak Dev Rev 1995 34 4 959 970

[B14] Akram M Khan FJ Health care services and government spending in Pakistan Pakistan Institute of Development Economics 2007 Working Paper Number 32

[B15] The World Bank Formal Survey 1999 The World Bank, In Monitoring and Evaluation: Some tools, methods and approaches. Washington D C: The World Bank

[B16] Mullahy J Econometric modeling of health care costs and expenditures: a survey of analytical issues and related policy considerations Med Care 2009 47 7 Suppl 1 S104 S108 19536020 10.1097/MLR.0b013e31819c9593

[B17] Matsaganis M Mitrakos T Tsakloglou P Modeling health expenditure at the household level in Greece Eur J Health Econ 2009 10 329 336 10.1007/s10198-008-0137-y 19037671

[B18] Rous JJ Hotchkiss DR Estimation of the determinants of household healthcare expenditure in Nepal with control for endogenous illness and provider choice Health Econ 2003 12 431 451 10.1002/hec.727 12759914

[B19] Tin Su T Pakhrel S Gbangou A Flessa S Determinants of household health expenditure on western institutional care Eur J Health Econ 2006 7 199 207 16673075 10.1007/s10198-006-0354-1

[B20] Okunade AA Suraratdecha C Benson DA Determinants of Thailand household healthcare expenditure: the relevance of permanent resources and other correlates Health Econ letters 2010 19 3 365 376 10.1002/hec.1471 19405046

[B21] Xu K Klavus J Kawabata K Evans DB Hanvoravongchai P Ortiz JP Zeramdini R Murray CJL Murray CJL, Evans DB Household health system contributions and capacity to Pay: definitional, empirical, and technical challenges Chapter 39 Health System Performance Assessment 2003 World Health Organization, Geneva 533 542

[B22] O’Donnell O van Doorslaer E Rannan-Eliya RP Somanathan A Adhikari SR Akkazieva B Harbianto D Garg CC Hanvoravongchai P Huq MN Karan A Leung GM Ng CW Pande BR Racelis R Tin K Tisayaticom K Trisnantoro L Zhang Y Zhao Y The incidence of public spending on healthcare: comparative evidence from Asia The World Bank Econ Rev 2007 21 1 93 123 10.1093/wber/lhl009

[B23] Gwatkin DR How much would poor people gain from faster progress towards the millennium development goals for health? The Lancet 2005 365 813 817 10.1016/S0140-6736(05)17992-6 15733726

